# Usefulness of 11C-Methionine Positron Emission Tomography for Differentiating Tumor Recurrence and Necrosis in Ethmoid Sinus Cancer With Intracranial Extension

**DOI:** 10.7759/cureus.90720

**Published:** 2025-08-22

**Authors:** Takehito Kishino, Takenori Miyashita, Yohei Ouchi, Junko Sogo, Hiroshi Hoshikawa

**Affiliations:** 1 Otolaryngology, Kagawa University, Kagawa, JPN

**Keywords:** chemoradiotherapy, fdg pet, met pet, mri, paranasal cancer

## Abstract

For head and neck cancer, treatment efficacy and recurrence are often assessed by morphological imaging methods such as CT and MRI. However, these techniques are sometimes ineffective for distinguishing qualitative changes in tumors following treatment. Here, we report the case of a 50-year-old male patient who received chemoradiotherapy for head and neck squamous cell carcinoma with intracranial extension. Following treatment, the patient’s symptoms subsided, and MRI at two and six months demonstrated tumor shrinkage. Six months after treatment, fluorodeoxyglucose (FDG) positron emission tomography (PET) revealed changes in the intracranial lesions, which were difficult to distinguish between tumor recurrence and necrosis. Consequently, we used 11C-methionine (MET) PET during follow-up. The findings from MET PET showed no evidence of disease recurrence; thus, the lesions were subsequently monitored for necrotic changes. This case suggests the potential utility of MET PET for differentiating recurrence from necrosis in head and neck cancers with intracranial extension. Further studies with larger cohorts are warranted to clarify its diagnostic accuracy and establish appropriate evaluation criteria.

## Introduction

When diagnosing head and neck cancer and evaluating treatment efficacy, morphological imaging techniques, such as computed tomography (CT) and magnetic resonance imaging (MRI), are sometimes insufficient for accurate assessment [1]. Positron emission tomography (PET) represents the most prominent qualitative diagnostic method, with fluorodeoxyglucose (FDG) PET being the modality most commonly applied in head and neck cancers. Several studies have evaluated the treatment efficacy of chemoradiotherapy using FDG PET [2,3].

Paranasal sinus cancer is a rare malignancy, accounting for approximately 3% of all head and neck cancers [4]. The majority of these tumors arise in the maxillary sinus, followed by the ethmoid sinus. About half of paranasal sinus malignancies are squamous cell carcinoma [5]. In primary tumors of the ethmoid sinus, locally advanced disease may extend into the anterior cranial fossa via the cribriform plate or into the orbit via the lamina papyracea.

In cases of ethmoid sinus carcinoma with intracranial extension treated with chemoradiotherapy, peritumoral edema or necrosis in the brain parenchyma may occur at the time of treatment response assessment. As a result, when lesions are located within the brain parenchyma, accurate evaluation using FDG PET may be challenging [6].

11C-methionine (MET) PET is a molecular imaging modality that utilizes radiolabeled methionine to assess amino acid metabolism in tissues [7]. Unlike FDG PET, which reflects glucose metabolism, MET PET provides more specific information on protein synthesis and cell proliferation [8]. This makes it particularly useful for evaluating brain tumors, where FDG PET may be limited due to physiological uptake in surrounding tissues such as the brain or inflamed regions.

Because MET is actively transported into tumor cells via amino acid transporters and incorporated into proteins, MET PET can provide better tumor delineation and help detect recurrence. In head and neck malignancies with intracranial extension, MET PET can offer clearer contrast between tumor tissue and surrounding brain parenchyma, enabling more accurate post-treatment assessment.

Additionally, MET PET is known for its lower background uptake in normal brain tissue, which enhances lesion detectability in central nervous system involvement. Despite its shorter half-life (20 minutes) compared to FDG, the higher specificity of MET often outweighs this limitation in selected cases.

Here, we present a case of ethmoid sinus cancer with intracranial extension in which MET PET was applied to distinguish recurrence from necrosis after chemoradiotherapy, and it appeared to be useful.

## Case presentation

**Figure 1:**
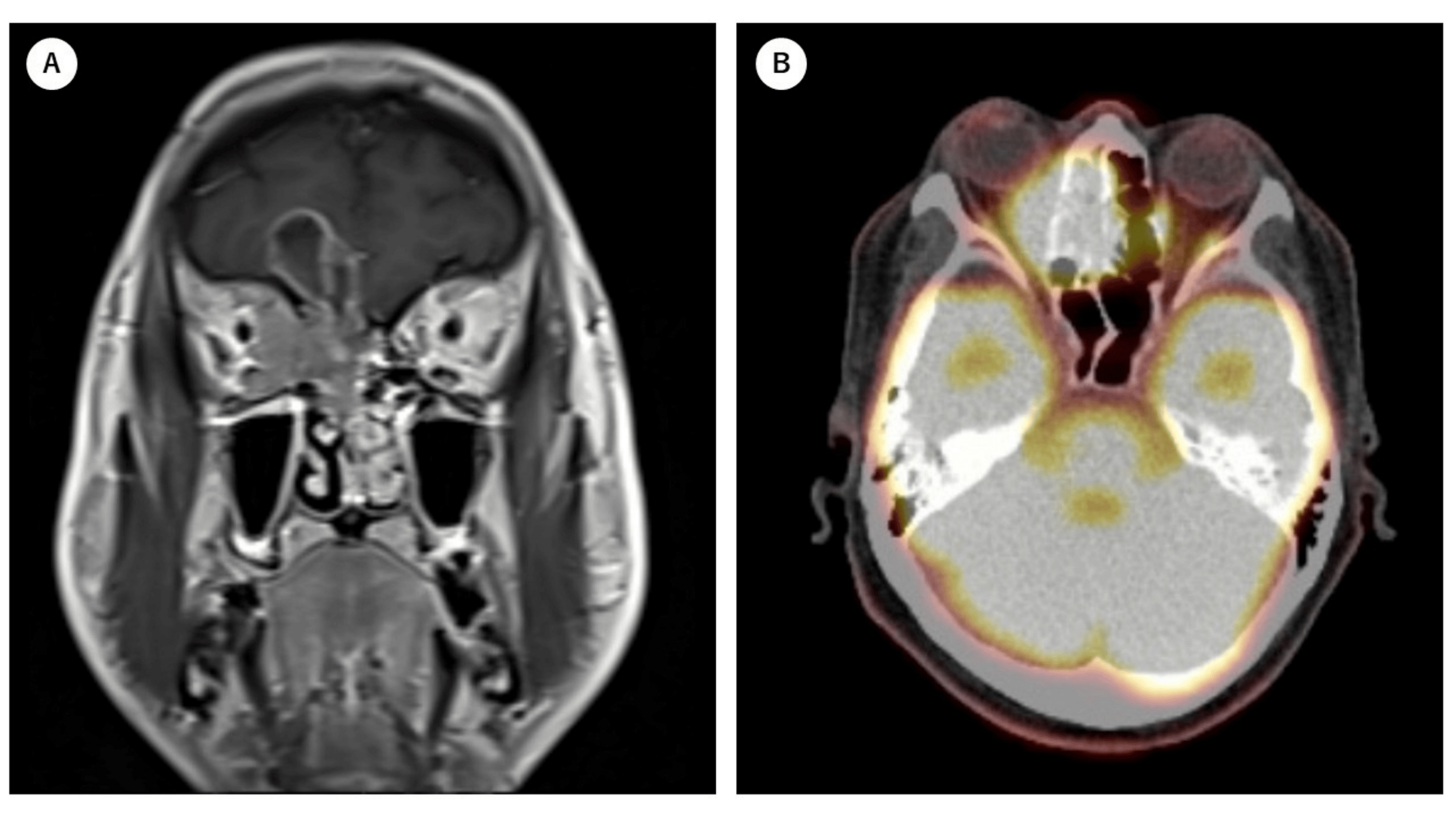
Pre-treatment MRI and FDG PET imaging A. MRI showing a T1-weighted tumor with gadolinium contrast enhancement in the right nasal cavity. The space-occupying lesion (SOL) with contrast enhancement at the edge of the right frontal lobe is shown. B. There is increased accumulation of FDG in the right paranasal cavity. FDG: fluorodeoxyglucose; PET: positron emission tomography

A 50-year-old man who presented with eye pain, diplopia, and hyposmia was referred to the Department of Otolaryngology of Kagawa University Hospital. A tumor was found in the right nasal cavity, which replaced the upper part of the middle turbinate. A biopsy confirmed the diagnosis of squamous cell carcinoma. MRI revealed a tumor extending from the right ethmoid sinus into the orbit and cranial cavity. FDG PET revealed FDG accumulation with a maximum standardized uptake (SUVmax) value of 14.0 in the same area (Figure 1), but no accumulation was observed in the cervical lymph nodes or other distant sites. Based on these findings, the patient was diagnosed with ethmoid sinus cancer, cT4bN0M0, Stage IVB.

After discussion with the Tumor Board of the hospital, intensity modulated radiation therapy (IMRT) in combination with cisplatin (CDDP) was initiated. Single doses of 2 Gy, which reached a total dose of 70 Gy of radiation, were administered; CDDP 80 mg/m2 was administered every three weeks during the treatment period. As renal function dropped after the first administration of CDDP, the dose was changed to 40 mg/m2 weekly for subsequent administrations, with a total of 200 mg/m2 of CDDP administered. The overall treatment duration was 49 days.

**Figure 2:**
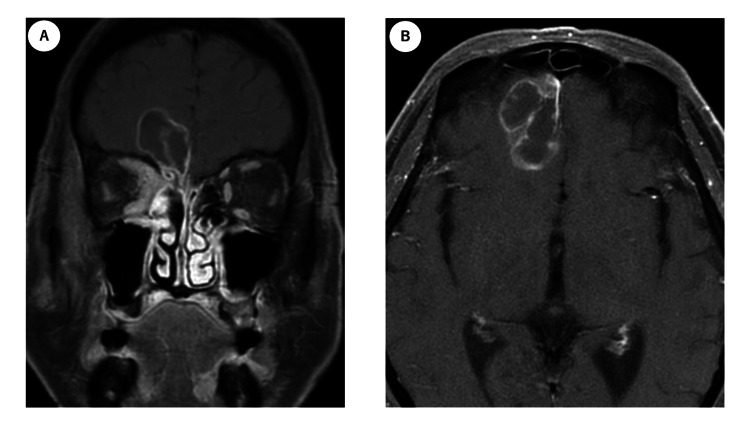
Post-treatment MRI after two months A, B: coronal and axial image The tumor of the paranasal cavity, shown in a T1-weighted image with gadolinium contrast enhancement, was significantly decreased. No noticeable changes were observed in the intracranial lesions.

A follow-up MRI was scheduled two months after treatment, as salvage surgery might be considered if residual disease was evident. Symptoms of eye pain and diplopia improved during treatment, and a two-month post-treatment MRI showed areas with contrast enhancement on T1-weighted gadolinium (T1W1Gd+) images in the nasal cavity, a space-occupying lesion (SOL) with low signal intensity on T1W1, and contrast enhancement at the margin in the right frontal lobe (Figure 2).

**Figure 3:**
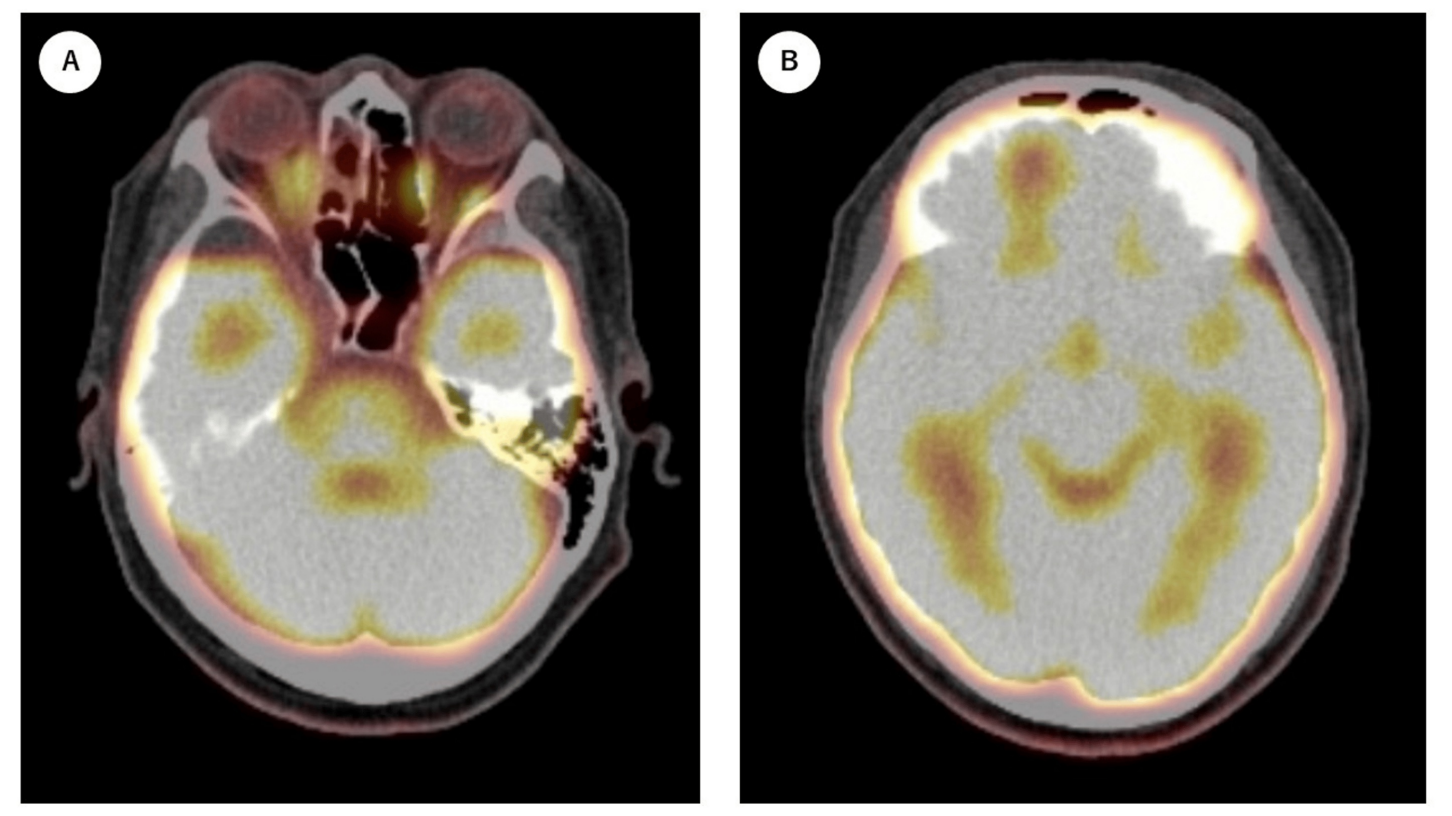
FDG PET imaging six months after completion of treatment. A. FDG accumulation in the right paranasal lesion has disappeared. B. The area surrounding the region of decreased FDG uptake was difficult to evaluate due to physiological accumulation in the brain. FDG: fluorodeoxyglucose; PET: positron emission tomography

Six months after treatment completion, MRI revealed that the areas with contrast enhancement in the paranasal and cranial cavities maintained a low signal intensity, suggesting a low-activity state. FDG PET was performed for qualitative evaluation, and abnormal FDG accumulation in the paranasal and orbital cavities had disappeared. The FDG accumulation, indicating the intracranial lesion in the right frontal lobe, also appeared to be reduced. However, the area surrounding the region of decreased FDG uptake was difficult to evaluate due to physiological accumulation in the brain (Figure 3).

**Figure 4:**
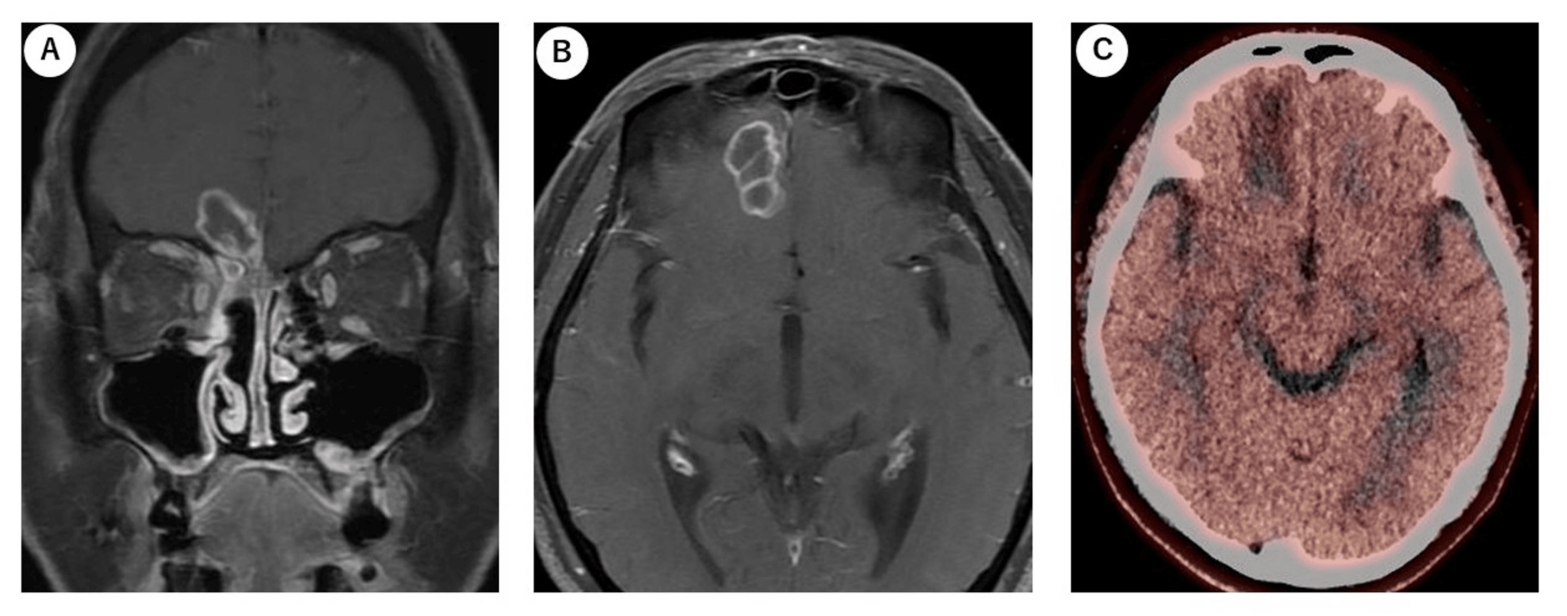
MRI and MET PET imaging after six months A, B: coronal and axial MRI images C: MET PET imaging. Very slight accumulation was observed with a mean standardized uptake (SUVmean) of 1.51 in the tumor and 1.4 in normal brain tissue. MET: 11C-methionine; PET: positron emission tomography

Following a neurosurgeon’s suggestion, MET PET was performed to differentiate between recurrence and necrosis of the intracranial lesion. The results showed very mild accumulation in the right skull base, with a tumor-to-normal tissue ratio (T/N) of 1.08 (Figure 4).

**Figure 5:**
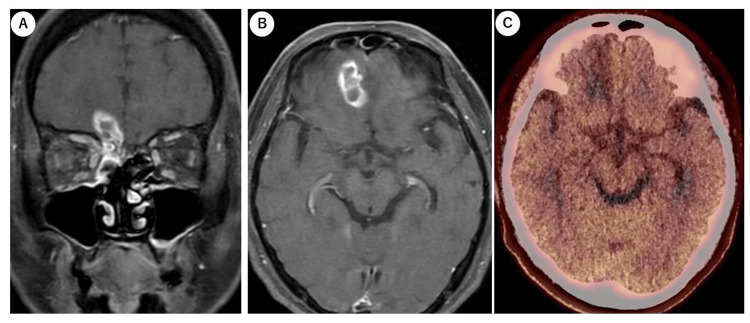
Final MRI and MET PET imaging A, B: coronal and axial MRI images. The most recent MRI findings indicated no lesions in the right paranasal cavity; however, the space-occupying lesion (SOL) in the cranial cavity showed increased contrast enhancement in some areas, and the edematous changes in the surrounding brain parenchyma had worsened. C: MET PET imaging. Mean standardized uptake (SUVmean) of 1.39 in the tumor and 1.07 in normal brain tissue was observed. MET: 11C-methionine; PET: positron emission tomography

Consequently, the lesion was considered to be controlled, and a follow-up MRI at six months was deemed sufficient. At one-year after treatment completion, MRI showed that the SOL with marginal contrast enhancement in the intracranial lesion had not changed in size. However, the thickness of the contrast-enhanced area had increased, and the surrounding brain parenchyma showed worsening edema, suggesting a recurrence of the lesion. To differentiate between recurrence and necrosis and to determine the necessity of therapeutic intervention, MET PET was repeated. Tumor mean standardized uptake (SUVmean) of 1.39, normal brain SUVmean of 1.07, and T/N ratio of 1.29 were obtained, with no evident disease progression, leading to a diagnosis of radiation necrosis (Figure 5).

Therapeutic intervention was considered unnecessary at this stage, and continued MRI follow-up after six months was planned.

## Discussion

Many reports have evaluated the treatment efficacy of chemoradiotherapy for head and neck cancer using FDG PET [1-3]. However, treatment efficacy is typically evaluated at least three months after treatment because, before that point, false positives may occur due to residual inflammation [4]. To exclude physiological FDG accumulation in inflammatory sites and brain or tonsil tissue, tracers reflecting nucleic acid metabolism, such as 3'-deoxy-3'-(18F) fluorothymidine (FLT) [9-11] and 4'-(Methyl-11C) thiothymidine (4DST) [12], are used to diagnose head and neck cancer and to determine treatment efficacy. However, standardized methods for evaluating head and neck cancer with intracranial spread have not yet been established.

In the field of neurosurgery, reports on the correlation between FLT or MET and brain tumor malignancy exist [13-15], and they demonstrate the usefulness of these markers in distinguishing between necrosis and other conditions. In brain tumors, the optimal T/N cutoff value of MET PET for determining tumor recurrence or necrosis was 1.40, with sensitivity and specificity values of 0.82 and 0.75, respectively [16]. In this study, MET PET was used to evaluate the efficacy of chemoradiotherapy in a patient with ethmoid sinus cancer that had progressed to the cranial cavity. The results showed no evidence of recurrence over two examinations spanning six months, leading to the conclusion that follow-up observation was sufficient.

Only a few reports have evaluated MET PET in head and neck cancers. Lindholm et al. examined 39 newly diagnosed cases and reported a median MET uptake of 9.0 (range, 4.0-18.8) [17]. Wedman et al. compared FDG and MET PET for laryngeal cancer after radiotherapy, reporting recurrence detection sensitivity and specificity of 77.3% and 56.0% for FDG PET versus 54.5% and 76.0% for MET PET [18]. Their analyses were qualitative. In our case, quantitative analysis using T/N ratios provided additional diagnostic value. However, whether this approach is appropriate requires further investigation.

A limitation of this case is the absence of pre-treatment MET PET, which prevented a comparison of pre- and post-treatment parameters. Such comparisons might have strengthened the interpretation. Performing pre-treatment MET PET could be considered in future cases of head and neck carcinoma with intracranial extension.

Taken together, this case highlights both the potential utility and the limitations of MET PET for assessing head and neck cancers with intracranial extension. While our findings support its role in differentiating recurrence from necrosis, larger studies are needed to validate diagnostic thresholds and establish standardized evaluation protocols.

## Conclusions

Conventional imaging modalities, such as MRI and FDG PET, have limitations in distinguishing between tumor recurrence and necrosis due to overlapping radiological findings and physiological FDG accumulation in the brain, where glucose metabolism is inherently high. In contrast, MET PET, which utilizes radiolabeled methionine--a substrate for protein synthesis--enables visualization of protein synthesis activity. Because MET PET exhibits low background uptake in normal brain parenchyma, it clearly demonstrated the absence of metabolically active tumor tissue in the present case of squamous cell carcinoma with intracranial extension, making it a valuable diagnostic tool. This case suggests the potential utility of MET PET, which enables an assessment of protein synthesis activity, in the evaluation of complex intracranial lesions following chemoradiotherapy, though further studies are required to confirm its role and refine evaluation criteria.
